# Association between major dietary patterns and obesity phenotypes in southwest China: baseline survey results from Hechuan

**DOI:** 10.3389/fnut.2024.1467025

**Published:** 2024-11-01

**Authors:** Wang Shaomei, Jing Dezhi, Li Mengfen, Duan Huaan, Ding Xianbin, Peng Juan, Li Xia, Zhu Yanfeng

**Affiliations:** ^1^Hechuan District Center Disease Control and Prevention, Chongqing, China; ^2^Department of Nutrition and Health Management, Chengdu Medical College, Chengdu, China; ^3^Chongqing Municipal Center for Disease Control and Prevention, Chongqing, China

**Keywords:** dietary pattern, obesity phenotype, metabolic syndrome, factor analysis, China

## Abstract

**Background:**

This study aimed to identify the main dietary patterns in Hechuan and clarify how they are associated with obesity phenotypes.

**Methods:**

A cross-sectional study was conducted based on a baseline survey of a general population cohort study in southwest China. A semi-quantitative food frequency questionnaire (FFQ) was used to investigate the dietary habits of the participants in the past year. Principal component analysis was conducted to identify the main dietary patterns, and multinomial logistic regression analysis was conducted to describe the association between the major dietary patterns and obesity phenotypes.

**Results:**

Three major dietary patterns were identified. The participants who followed the wheaten food dietary pattern had a higher likelihood of having metabolically normal obesity (MHO) (odds ratio (OR) 1.05, 95% confidence interval (CI) 1.02–1.08), metabolically abnormal normal weight (MUNW) (OR 1.08, 95%CI 1.00–1.16), and metabolically abnormal obesity (MUO) (OR 1.07, 95%CI 1.04–1.11). Specifically, those with the highest wheaten food dietary pattern were 1.60 times more likely to have MHO (OR 1.60, 95%CI 1.25–2.05), 2.62 times more likely to have MUNW (OR 2.62, 95%CI 1.28–5.37), and 2.01 times more likely to have MUO (OR 2.01, 95%CI 1.51–2.69) than those with the lowest wheaten food dietary pattern.

**Conclusion:**

The wheaten food dietary pattern may increase the risk of obesity and metabolic abnormalities. Therefore, timely interventions should be carried out for this group of people.

## Introduction

Obesity is a multifactorial, relapsing, and chronic disease (1). In 2015, global obesity reached 107.7 million in children and 603.7 million in adults (2). Since the reform and opening-up policy, the prevalence of obesity in China has been increasing at an alarming rate (3). In China, from 2014 to 2018, the prevalence of overall obesity and abdominal obesity was 15.8 and 37.6% in women and 15.0 and 36.3% in men, respectively (4). In 2022, obesity and central obesity among adults over 18 years old in Hechuan were 16.16 and 33.51%, respectively (5). Obesity is linked to a range of diseases and complications, which can complicate obesity prevention and management (6–10). The traditional definition of obesity only considers the body mass index (BMI). However, the “obesity paradox” tends to emerge in this classification (11, 12). Therefore, it is unacceptable to evaluate the risk of obesity on health only based on the BMI (13). An alternative approach is to combine the BMI and metabolic syndrome (MetS) to identify heterogeneity in obesity. Patients can be divided into four phenotypes: metabolically healthy normal weight (MHNW), metabolically normal obesity (MHO), metabolically abnormal normal weight (MUNW), and metabolically abnormal obesity (MUO). The term “metabolic health” here refers to having no presence of MetS (14). The prevalence of MHO, MUNW, and MUO in China has been reported to be 3.9, 18.1, and 9.8%, respectively (15). Different types of obesity have varying impacts on health. Therefore, it is crucial to categorize obese patients based on their phenotype, identify potential risk factors, and intervene at the right time to reduce the incidence of obesity.

Lifestyle intervention has been regarded as the cornerstone of obesity management (16). Except for individual nutrients, more attention is given to overall dietary patterns and their impact on obesity and cardiovascular metabolism (17). It is important to conduct an overall dietary pattern analysis to better understand how different dietary patterns contribute to obesity and MetS. However, there are limited studies on the relationship between dietary patterns and obesity in Hechuan, Chongqing, China. A general population cohort study in southwest China (multi-ethnic cohort study) was conducted in 2017 (18). Hechuan, as a subcohort, completed a baseline survey of local residents in 2018. Based on the baseline data, this study aimed to (1) identify the main dietary patterns of residents in Hechuan; (2) describe the socio-demographic and lifestyle characteristics of different obesity phenotypes in Hechuan; and (3) investigate the association between major dietary patterns and obesity phenotypes and identify factors influencing obesity phenotypes.

## Methods

Data were derived from the baseline survey of the 2018 multi-ethnic cohort study conducted in Hechuan. A multi-stage stratified random sampling method was used to select residents aged 30–79 years in Hechuan, Chongqing, as the research participants. We have provided the STROBE checklist in the supplementary materials.

Previous studies have elucidated comprehensive methodological and research protocols (19). Using PASS software, the minimum sample size was calculated to be 500. A total of 3,009 residents were included in the cohort. All participants completed a questionnaire, anthropometric measurements, and laboratory examinations. The Medical Ethics Review Committee of Sichuan University and the local review committee approved the research protocol. All respondents participated voluntarily and signed an informed consent form. A total of 2,929 participants were included in the analysis after excluding those with a BMI of less than 18.5 kg/m^2^. Trained and qualified investigators used tablet computers to conduct the electronic questionnaire survey for all participants in the form of face-to-face interviews. This questionnaire was developed for the multi-ethnic cohort study and was validated (20). Standard methods were used to measure height, weight, waist circumference (WC), hip circumference, and blood pressure. Mean systolic blood pressure (SBP) and mean diastolic blood pressure (DBP) are the average of three consecutive measurements, and the BMI (kg/m^2^) is calculated by dividing weight (kg) by height (m) squared. The AU5800 automatic biochemical analyzer (Beckman Coulter Co, LTD) was used to test the biological samples.

The dietary habits of the participants were investigated using a semi-quantitative food frequency questionnaire (FFQ) to obtain the average consumption amount of each person for every food. Based on the Kaiser–Meyer–Olkin (KMO) result of 0.66 and the Bartlett’s test of sphericity (BTS) result of *p* < 0.001, it appeared that the data were appropriate for factor analysis. Eigenvalue >1 was considered the criterion for the inclusion of common factors. Three major dietary patterns were identified, namely “white meat and egg dietary pattern,” “rice and red meat dietary pattern,” and “wheaten food dietary pattern.”

According to the Expert Consensus on Obesity Prevention and Control of Chinese Residents (21), a BMI of less than 18.5 kg/m^2^ is considered underweight, 18.5 to 23.9 kg/m^2^ is considered normal weight, 24.0 to 27.9 kg/m^2^ is considered overweight, and 28.0 kg/m^2^ or above is considered obesity. According to the Unified Joint Scientific Statement (22), those who meet three or more of the following criteria are diagnosed with MetS: (1) abdominal obesity: men WC ≥90 cm and women WC ≥85 cm; (2) hyperglycemia: fasting plasma glucose (FPG) ≥6.1 mmol/L; (3) elevated blood pressure: SBP > 130 and/or DBP > 85 mmHg; (4) triglyceride (TG) ≥1.70 mmol/L; and (5) high-density lipoprotein-cholesterol (HDL-C) < 1.0 mmol/L in men and < 1.3 mmol/L in women. According to the BMI and metabolic status, the study participants could be divided into four obesity phenotypes: MHNW, MHO, MUNW, and MUO.

Continuous variables were expressed as mean ± standard deviation. Categorical variables were expressed as *n* (%). An ANOVA was used for the continuous variables, while a chi-square test was used for the categorical variables. The pairwise comparison was corrected using the Bonferroni method (23). The association of the major dietary patterns with the obesity phenotypes was quantified by multinomial logistic regression analysis, with MHNW as the reference group. We adjusted for the following confounding factors: age, gender, education, household income, marital status, symptoms of insomnia, symptoms of anxiety, symptoms of depression, total daily energy intake, smoking, and drinking. A linear trend test was performed by entering the median of each quartile into the model as a continuous variable (20). Statistical analysis was performed using the R Project for Statistical Computing version 4.2.3 (Vienna, Austria).

## Results

Among the four phenotypes, the MHO phenotype accounted for 37.97% of the total population and 61.74% of the overweight/obese population. The MHNW phenotype accounted for 35.78% of the total population and 92.90% of the normal-weight population. The MUO phenotype accounted for 23.52% of the total population and 38.26% of the overweight/obese population. The MUNW phenotype accounted for 2.73% of the total population and 7.09% of the normal-weight population. The population-based sociological characteristics of the different phenotypes are shown in Table 1. MHNW was the reference group, while the other three groups were different in age, and the individuals with unhealthy metabolism were more likely to be elderly. Regardless of metabolism, the obese individuals were more likely to be those with junior high school education or below and premenopausal women. The MUO group was more likely to include widows, smokers, spicy eaters, individuals with depressive symptoms, and those with a family history of cardiovascular disease. The MHO group was more likely to include those with low income and beverage consumers.

**Table 1:** Demographic characteristics of different obesity phenotypes.

Characteristics	MHNW	MHO	MUNW	MUO	*p*-value
*N* (%)	1,048 (35.78)	1,112 (37.97)	80 (2.73)	689 (23.52)	<0.001
Gender (%)					<0.001
Men (%)	382 (36.45)	**481 (43.26)**	30 (37.50)	**361 (52.40)**	
Women (%)	666 (63.55)	**631 (56.74)**	50 (62.50)	**328 (47.61)**	
Age	49.73 ± 11.70	**51.07 ± 10.87**	**59.59 ± 9.53**	**56.10 ± 11.03**	<0.001
Marital status (%)					<0.001
Married/cohabiting (%)	939 (89.60)	1,024 (92.09)	67 (83.75)	**611 (88.68)**	
Separated/divorced (%)	59 (5.63)	45 (4.05)	6 (7.50)	**26 (3.77)**	
Widowed (%)	39 (3.72)	33 (2.97)	7 (8.75)	**50 (7.26)**	
Spinsterhood (%)	11 (1.05)	10 (0.90)	0 (0.00)	**2 (0.29)**	
Education (%)					<0.001
No schooling (%)	74 (7.06)	**105 (9.44)**	9 (11.25)	**94 (13.64)**	
Primary school (%)	298 (28.44)	**377 (33.90)**	30 (37.50)	**206 (29.90)**	
Middle school (%)	358 (34.16)	**362 (32.55)**	20 (25.00)	**233 (33.82)**	
High school (%)	167 (15.94)	**160 (14.39)**	15 (18.75)	**97 (14.08)**	
College/university (%)	151 (14.41)	**108 (9.71)**	6 (7.50)	**59 (8.56)**	
Household income (Yuan/year, %)					0.05
<59,999	640 (61.07)	**743 (66.82)**	56 (70.00)	435 (63.13)	
60,000–99,999	207 (19.75)	**199 (17.90)**	18 (22.50)	140 (20.32)	
100,000–199,999	164 (15.65)	**148 (13.31)**	5 (6.25)	94 (13.64)	
≥200,000	37 (3.53)	**22 (1.98)**	1 (1.25)	20 (2.90)	
Agricultural work (%)	236 (22.52)	298 (26.80)	21 (26.25)	169 (24.53)	0.14
Smoke (%)	236 (22.52)	298 (26.80)	21 (26.25)	169 (24.53)	<0.001
Drinks (%)	564 (53.82)	602 (54.14)	34 (42.50)	395 (57.33)	0.07
Beverages (%)	19 (1.81)	**40 (3.60)**	2 (2.50)	14 (2.03)	0.05
Dietary supplement intake (%)	174 (16.60)	191 (17.18)	14 (17.50)	112 (16.26)	0.95
Spicy food eaters (%)	892 (85.11)	928 (83.45)	65 (81.25)	591 (85.78)	0.43
Peppery food eaters (%)	890 (84.92)	936 (84.17)	62 (77.50)	**612 (88.82)**	<0.001
Total energy intake (kcal/day)	1909.32 (590.57)	1915.38 (617.25)	1816.30 (612.55)	1935.36 (613.60)	0.57
Physical activity (MET-hours/day)	2.15 (3.38)	2.28 (3.35)	3.17 (4.52)	2.66 (3.66)	0.07
Insomnia symptoms (%)	436 (41.60)	479 (43.08)	38 (47.50)	328 (47.61)	0.08
Depressive symptoms (%)	297 (28.34)	305 (27.43)	30 (37.50)	**227 (32.95)**	0.02
Anxiety symptoms (%)	344 (32.82)	353 (31.74)	26 (32.50)	234 (33.96)	0.81
Family history of cardiovascular disease (%)	61 (5.82)	51 (4.59)	3 (3.75)	**55 (7.98)**	0.02
Menopausal status in women (%)					<0.001
Perimenopausal	393 (59.01)	**331 (52.46)**	**8 (16.00)**	**56 (17.07)**	
Postmenopausal	44 (6.61)	**40 (6.34)**	**0 (0.00)**	**17 (5.18)**	
Premenopausal	229 (34.38)	**260 (41.20)**	**42 (84.00)**	**255 (77.74)**	

Bold numbers in the table represent *p* < 0.05. MET, metabolic equivalent of task.

The MHO, MUNW, and MUO phenotypes had lower mean concentrations of HDL cholesterol compared to the MHNW phenotype. However, other metabolic parameters in the three phenotypes were higher than in the MHNW phenotype. The proportion of abnormalities in the biochemical parameters defining metabolic health was higher in the MHO, MUNW, and MUO phenotypes than in the MHNW phenotype (Table 2).

**Table 2:** Metabolic status of different obesity phenotypes.

Characteristics	MHNW	MHO	MUNW	MUO	*p*-value
Metabolic parameters					
BMI (kg/m^2^)	22.03 (1.35)	**26.32 (1.97)**	**22.63 (1.16)**	**28.19 (2.52)**	**< 0.001**
Glucose (mmol/l)	5.19 (0.83)	5.30 (0.91)	**6.88 (2.16)**	**6.38 (1.91)**	**< 0.001**
SBP (mmHg)	125.91 (18.47)	**131.32 (18.52)**	**145.03 (16.53)**	**145.42 (17.83)**	**< 0.001**
DBP (mmHg)	73.59 (10.40)	**76.95 (10.62)**	**80.77 (11.73)**	**82.98 (11.14)**	**< 0.001**
Triglycerides (mmol/l)	1.12 (0.75)	**1.32 (0.72)**	**2.49 (1.53)**	**2.30 (1.37)**	**< 0.001**
HDL-C (mmol/L)	1.79 (0.42)	**1.61 (0.38)**	**1.34 (0.41)**	**1.30 (0.36)**	**< 0.001**
Abdominal obesity defined by WHR (%)^a^	341 (32.54)	**713 (64.12)**	**59 (73.75)**	**637 (92.45)**	**< 0.001**
Abdominal obesity defined by WC (%)^b^	18 (1.72)	**378 (34.00)**	**16 (20.00)**	**569 (82.58)**	**< 0.001**
Elevated triglycerides (%)^c^	102 (9.73)	**183 (16.46)**	**56 (70.00)**	**453 (65.75)**	**< 0.001**
Elevated BP (%)^d^	339 (32.35)	**493 (44.33)**	**71 (88.75)**	**585 (84.91)**	**< 0.001**
Lowered HDL-cholesterol (%)^e^	51 (4.87)	**76 (6.83)**	**38 (47.50)**	**266 (38.61)**	**< 0.001**
Elevated glucose (%)^f^	136 (12.97)	**179 (16.10)**	**65 (81.25)**	**442 (64.15)**	**< 0.001**
Other variables					
LDL-C (mmol/l)	2.65 (0.80)	**2.87 (0.84)**	2.79 (0.89)	**2.94 (0.85)**	**< 0.001**
Total cholesterol (mmol/L)	4.97 (0.94)	**5.09 (0.97)**	**5.27 (1.06)**	**5.29 (0.99)**	**< 0.001**
UA (μmol/L)	283.81 (71.88)	**307.27 (80.52)**	**312.28 (89.60)**	**349.32 (87.56)**	**< 0.001**

Bold numbers in the table represent *p* < 0.05.

a Central obesity was defined by WHR: ≥0.9 in the men and ≥ 0.85 in the women.

b Central obesity was defined by waist circumference: ≥90 cm in the men and ≥ 85 cm in the women.

c Elevated triglycerides: ≥1.7 mmol/L.

d Elevated BP: SBP ≥ 130 mmHg or DBP ≥ 85 mmHg.

e Lowered HDL cholesterol: <1.0 mmol/L in the men and < 1.3 mmol/L in the women.

f Elevated glucose: ≥5.6 mmol/L.

Three major dietary patterns in Hechuan were identified by principal component analysis. The first dietary pattern was named “white meat and egg dietary pattern,” which mainly included poultry (0.56), eggs (0.53), sea/aquatic products (0.50), dairy products (0.48), and fresh fruits (0.47). The second dietary pattern was named “rice and red meat dietary pattern,” which mainly included rice (0.55) and red meats (0.46). The third dietary pattern was named “wheaten food dietary pattern,” which mainly included wheaten food (0.83).

**Figure 1:**
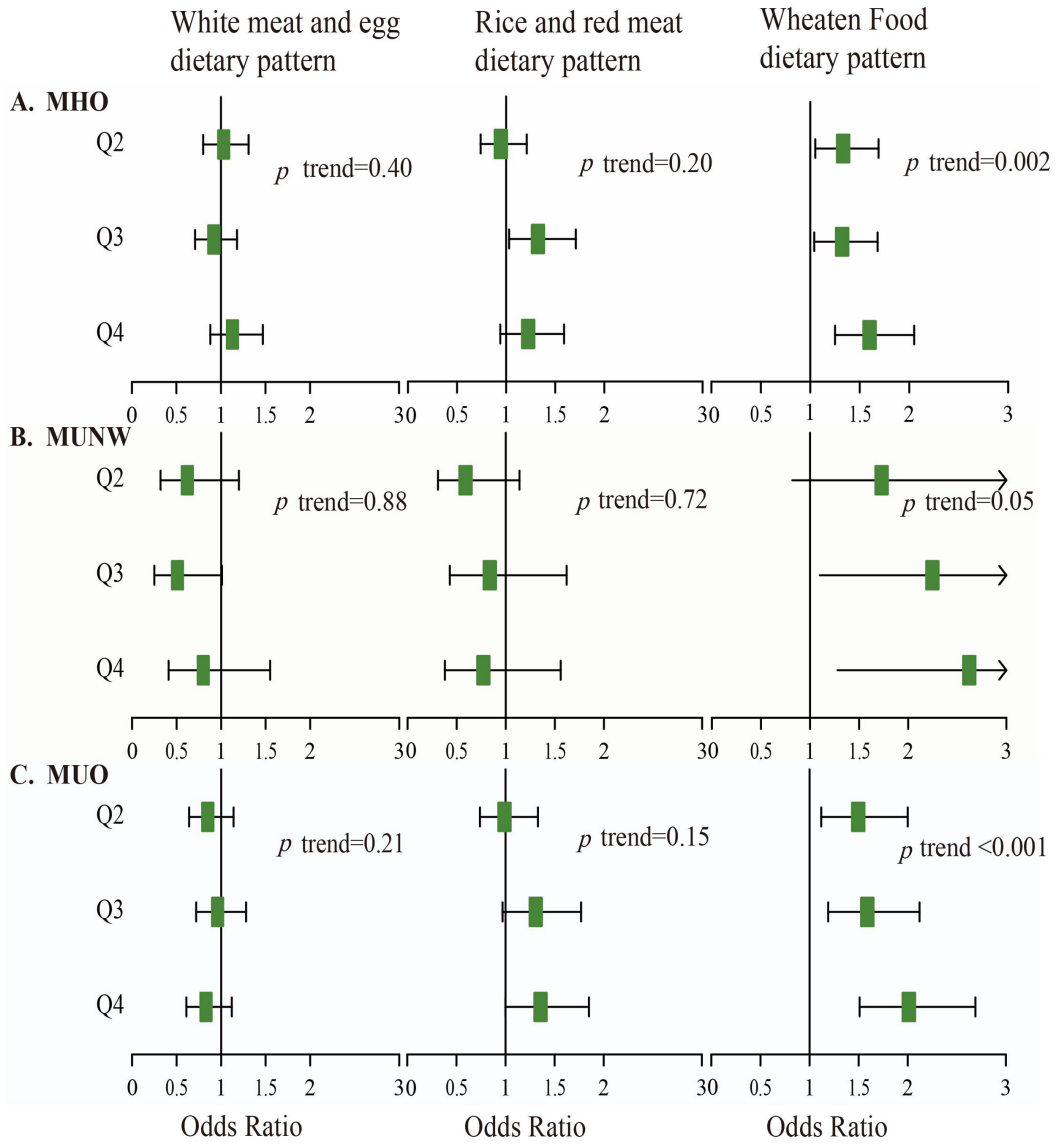
Association of three main dietary patterns with the MHO, MUNW, and MUO phenotypes. All models were adjusted for the confounding factors, with MHNW as the reference group. **(A)** Represents the correlation between the MHO phenotype and the three dietary patterns. **(B)** Represents the correlation between the MUNW phenotype and the three dietary patterns. **(C)** Represents the correlation between the MUO phenotype and the three dietary patterns.

Multinomial logistic regression analysis was used to detect the association between the three major dietary patterns and the obesity phenotypes (Figure 1). After correcting for the confounding factors mentioned above, it was found that the more the participants followed the wheaten food dietary pattern, the higher the likelihood of having MHO (odds ratio (OR) 1.05, 95% confidence interval (CI) 1.02–1.08), MUNW (OR 1.08, 95%CI 1.00–1.16), and MUO (OR 1.07, 95%CI 1.04–1.11). Specifically, those with the highest wheaten food dietary pattern were 1.60 times more likely to have MHO (OR 1.60, 95%CI 1.25–2.05), 2.62 times more likely to have MUNW (OR 2.62, 95%CI 1.28–5.37), and 2.01 times more likely to have MUO (OR 2.01,95%CI 1.51–2.69) than those with the lowest wheaten food pattern. The participants adhering to the wheaten food dietary pattern were at a higher risk of developing obesity, experiencing metabolic abnormalities, and having a combination of metabolic abnormalities and obesity. In addition, no significant association was observed between the white meat and egg dietary pattern and the white rice and red meat dietary pattern with the obesity phenotypes.

## Discussion

The prevalence of overweight/obesity in Hechuan was 61.49% (1801/2929), which is higher than the average level in China (42.8%) (24). Studies have shown that smoking, insufficient physical activity, unhealthy diet, and drinking increase the risk of obesity (25, 26). In Hechuan, the rates of smoking, drinking, and insufficient physical activity were 29.84, 54.07, and 12.79%, respectively (27). Apart from smoking and insufficient physical activity, the rate of drinking was higher than the national average level (28–30). Alcohol is a beverage with high calories. Each gram (g) of alcohol provides 7 kilocalories (kcal), which is slightly lower than that of fats (9 kcal/g) but nearly twice the amount of calories provided by carbohydrates or proteins (both contain 4 kcal/g) (31, 32). Moreover, alcohol can hinder the oxidative decomposition of fat, causing fat to accumulate in the liver and abdomen and leading to obesity (33, 34). Hechuan is popular for its Sichuan cuisine, which is characterized by abundant oil and salt, resulting in excessive intake of fat and sodium and eventually leading to obesity. Hechuan is a less developed city located in the southwest of China. The *per capita* disposable income of the residents is 39,286 yuan, and the Engel coefficient is 30.3% (35), which is higher than the national average level of 29.8% (36). Compared with the rest of the country, there may be a higher proportion of residents in Hechuan inclined to buy cheaper and unhealthier foods, such as fast foods, which results in excessive intake of fat and causes obesity.

Among the four obesity phenotypes, the prevalence of MHO and MUNW was 37.97 and 2.73%, respectively, which is similar to the results of previous studies (37–41). However, the prevalence of MUO was found to be higher at 23.52% compared to previous research results (15, 42, 43). Studies have shown that overweight, obesity, high-fat diet, and insufficient physical activity are the risk factors for MetS (44). Overweight and obesity can promote the development of MetS. The awareness rates of the recommended intakes of salt and oil in the Dietary Guidelines for Chinese Residents among the residents in Hechuan are quite low, at 24.50 and 15.45%, respectively, which may lead to excessive fat and sodium intakes (5). People with low educational levels have a higher BMI and WC, and there is a negative correlation with obesity (45). The average years of education for the population aged 15 and above in Hechuan are 9.21 years (46), which are lower than the national average level (9.91 years) (47). The high prevalence of MUO may be due to the fact that people with low educational levels do not pay enough attention to obesity and cannot intervene in time, as well as the lack of standardized management. When obese patients have normal metabolism, they are more likely to be overlooked, which can lead to the development of abnormal metabolism.

Compared with MHNW, the MHO phenotype has a higher proportion of men (43), older age (48), lower education (49), lower household income (50, 51), and a greater proportion of people who like to drink beverages (52). Perhaps due to a higher proportion of men having an adverse lifestyle compared to women, the smoking and drinking rates of men (74.30 and 53.80%) were significantly higher than those of women (3.20 and 12.20%) (28). The obesity prevalence shows a rapid increase among people over 25 years old and peaks at between 45 and 74 years old (53). Compared with MHNW, MUO had a higher proportion of individuals with low literacy, widows (54), smokers (55), individuals with depressive symptoms (56), and individuals with a family history of cardiovascular disease (43). The MUNW phenotype primarily included older populations (43, 57). These results are consistent with previous studies.

The metabolic parameters of the individuals with the other three obesity phenotypes tended to be less healthy compared to those of the individuals with the MHNW phenotype. Even under the premise of metabolic health, the metabolic parameters of the obese patients were more unhealthy than those of the non-obese patients, which is consistent with the results of previous studies (15, 58). Specifically, the MUNW and MUO phenotypes had a higher risk of chronic disease, cardiovascular events, and all-cause mortality when compared to the MHNW phenotype (37, 43, 59, 60). Thus, it is evident that in the efforts directed toward obesity prevention and control, attention should be paid to individuals with high-risk factors, such as the elderly, smokers, and those with low education levels. In addition, attention should be given to the promotion and education regarding obesity prevention and control, and the popularization of health knowledge should be strengthened to transform high-risk groups into individuals with healthy lifestyles. The government and medical institutions should intervene and manage obese patients in advance, establish a set of effective obesity management models, establish public facilities suitable for residents to engage in physical exercise, create a safe and hygienic eating environment, reduce the prevalence of obesity, reduce metabolic abnormalities, reduce the incidence of obesity complications, and reduce the burden of the disease. In addition, we also observed that a considerable proportion of the participants with normal weight, as determined by the BMI, had abdominal obesity, and an increasing number of studies have shown that visceral fat is closely related to the development of cardiovascular diseases (61–64). Therefore, it is essential to keep an eye on individuals who may appear healthy but have a high risk of developing cardiovascular disease due to the presence of visceral fat. Meanwhile, it is crucial to focus on high-risk populations and prompt them to adopt a healthy lifestyle to reduce the risk of obesity.

Our findings suggested that the wheaten food dietary pattern is strongly associated with the MHO, MUNW, and MUO phenotypes, which is consistent with previous research (65–67). Carbohydrates, which are a major component of the wheaten food dietary pattern, and high carbohydrates are positively correlated with obesity (68). The potential explanation for the increased risk of carbohydrate-related obesity is that carbohydrate intake may alter lipid profiles, such as increased triglycerides and/or decreased HDL cholesterol, leading to increased obesity (69). In addition, experiments based on animal models have shown that wheat gluten, a protein, promotes weight gain by reducing heat production and energy expenditure (70). Most of the time, noodles are used as the main course of a meal, with almost no vegetables, little meat, more oil, and heavy Sichuan pepper and other spices, such as “Chongqing noodles” (71–73), which is quite popular in Hechuan. However, no significant association was observed between other dietary patterns and the obesity phenotypes. This may require further investigation with larger sample sizes and more diverse data, and the potential association may need to be further explored.

In summary, the prevalence of MHO and MUNW in Hechuan was found to be at the average level, while the prevalence of MUO was found to be higher than that of other studies. Their negative effects on health, especially on metabolism, should not be overlooked. The risk factors of each metabolic phenotype should be taken into account, and classified interventions should be carried out for high-risk populations to reduce the incidence of obesity, complications, and the burden of diseases. The wheaten food dietary pattern was positively correlated with the MHO、MUNW and MUO phenotypes. Therefore, it is necessary to follow the 2022 Dietary Guidelines for Chinese Residents (74) to achieve a balanced and healthy diet, as well as a healthy lifestyle.

The collection of the data from a multi-ethnic cohort study and the selection of the participants through multi-stage stratified random sampling were considered the strengths of the study. However, there are also some limitations to this study, which include a small sample size and no follow-up data. In addition, the study’s ability to infer causality is limited due to the natural defects of cross-sectional research. Moreover, the dietary data were collected using the simplified FFQ scale to recall the participants’ eating habits, which could only provide a rough estimate of the food intake and led to recall bias. However, the investigation tried to control this bias by using standard bowls, spoons, and other tools to assist in the investigation of quantifying food. Future studies with larger population sample sizes and follow-up data are needed to further analyze the association between dietary patterns and obesity phenotypes while overcoming the limitations of causal inference in cross-sectional studies.

## Conclusion

In conclusion, different obesity phenotypes vary with demographic characteristics and metabolic parameters. In Hechuan, we identified three main dietary patterns and found that the wheaten food dietary pattern is positively correlated with the MHO, MUNW, and MUO phenotypes. The more the wheaten food dietary pattern was followed, the higher the risk of obesity and metabolic abnormalities was observed, while no correlation was observed between the other two dietary patterns and the obesity phenotypes. Therefore, in view of the two aspects of the obesity phenotypes, focus should be placed on the risk factors of obesity and MetS. It is crucial to take into account both socio-demographic risk factors and dietary risk factors when it comes to understanding and addressing obesity. Medical institutions should actively disseminate health knowledge to the masses so that they can choose healthy lifestyles, choose healthy foods according to dietary guidelines, and avoid excessive intake of fats and carbohydrates. The government should pay more attention to obesity and establish a set of effective obesity management models to effectively decrease the incidence of obesity and metabolic abnormalities and its complications, ultimately reducing the burden of the disease.

## Data Availability

The raw data supporting the conclusions of this article will be made available by the authors, without undue reservation.
